# Vivaria housing conditions expose sex differences in brain oxidation, microglial activation, and immune system states in aged hAPOE4 mice

**DOI:** 10.1007/s00221-023-06763-x

**Published:** 2024-01-11

**Authors:** E. M. Reyes-Reyes, J. Brown, M. D. Trial, D. Chinnasamy, J. P. Wiegand, D. Bradford, R. D. Brinton, K. E. Rodgers

**Affiliations:** 1 Center for Innovation in Brain Science, University of Arizona, 1230 N. Cherry Ave, PO Box 210242, Tucson, AZ 85721-0242 USA; 2 Department of Pharmacology, College of Medicine, University of Arizona, Tucson, AZ USA

**Keywords:** Alzheimer’s disease, Apolipoprotein, Sex differences, Immune status, Housing and food sterility

## Abstract

Apolipoprotein E ε4 allele (APOE4) is the predominant genetic risk factor for late-onset Alzheimer’s disease (AD). APOE4 mouse models have provided advances in the understanding of disease pathogenesis, but unaccounted variables like rodent housing status may hinder translational outcomes. Non-sterile aspects like food and bedding can be major sources of changes in rodent microflora. Alterations in intestinal microbial ecology can cause mucosal barrier impairment and increase pro-inflammatory signals. The present study examined the role of sterile and non-sterile food and housing on redox indicators and the immune status of humanized-*APOE4* knock-in mice (hAPOe4). hAPOE4 mice were housed under sterile conditions until 22 months of age, followed by the transfer of a cohort of mice to non-sterile housing for 2 months. At 24 months of age, the redox/immunologic status was evaluated by flow cytometry/ELISA. hAPOE4 females housed under non-sterile conditions exhibited: (1) higher neuronal and microglial oxygen radical production and (2) lower CD68^+^ microglia (brain) and CD8^+^ T cells (periphery) compared to sterile-housed mice. In contrast, hAPOE4 males in non-sterile housing exhibited: (1) higher MHCII^+^ microglia and CD11b^+^CD4^+^ T cells (brain) and (2) higher CD11b^+^CD4^+^ T cells and levels of lipopolysaccharide-binding protein and inflammatory cytokines in the periphery relative to sterile-housed mice. This study demonstrated that sterile vs. non-sterile housing conditions are associated with the activation of redox and immune responses in the brain and periphery in a sex-dependent manner. Therefore, housing status may contribute to variable outcomes in both the brain and periphery.

**Supplementary Information:**

The online version contains supplementary material available at 10.1007/s00221-023-06763-x.

## Introduction

Alzheimer's disease (AD) is the most common neurodegenerative disease, whose classical phenotype is prominent long-term memory deficits that progress into dementia. Pathophysiological hallmarks of AD include extracellular amyloid-beta (Aβ) plaques, intracellular neurofibrillary tau tangles, and neuroinflammation (Gholami 2023; Tzioras et al. 2023). Currently, AD remains incurable; however, treatments are available that can alleviate symptoms and enhance the quality of life for those affected (Scheltens et al. 2021). The elucidation of cellular and molecular mechanisms contributing to AD pathogenesis is crucial for identifying new therapeutic targets (Gholami 2023). Mechanisms such as oxidative damage and systemic inflammation may accelerate the progression of AD (Chen and Zhong 2014; Bello-Corral et al. 2023). Therapeutic interventions, including anti-inflammatory drugs and antioxidant supplements, have been observed to ameliorate symptoms or slow cognitive decline in AD patients (Fillit et al. 1986; Ozben and Ozben 2019; Collins et al. 2022).

The APOE4 polymorphism of apolipoprotein E (APOE) is the largest genetic risk factor for late-onset Alzheimer’s disease (LOAD). APOE is a lipid transporter that regulates cholesterol metabolism and facilitates the clearance of Aβ from the brain. APOE4 represents a loss of function in lipid redistribution to neurons for myelin maintenance and Aβ clearance (Kanekiyo et al. 2014). Nonetheless, given that only 50–60% of AD patients possess the APOE4 allele, increasing evidence points to a range of modifiable lifetime risk factors that can exacerbate AD risk. These include lifestyle choices such as diets high in fat, early-life stress, sleep disturbances, environmental pollution, and socio-economic challenges involving poor nutrition, inadequate healthcare, continuous stress, limited education, and insufficient sanitation. These factors may intensify the impact of non-modifiable AD risk factors such as age, gender, and APOE genotype. (Altmann et al. 2014; Zhang et al. 2021; Rosselli et al. 2022).

Neuroinflammatory responses can be triggered by factors confined within the central nervous system (CNS) and/or by systemic influences originating externally to the CNS. Recent research on the gut–brain axis has highlighted the role of the microbiome in Alzheimer’s etiology (Kesika et al. 2021). Barrier and non-barrier housing are commonly used systems in animal research facilities but are rarely reported. Barrier housing systems control the microbiological status of the animals (health status A), while non-barrier housing is a clean and hygienic environment, but not strictly microbiologically controlled (health status D). Non-barrier housing uses non-sterilized bedding, which has been reported as a source of contamination and modifies gut and cecum microbiota. Mice on non-sterile bedding for three months showed a higher total number of microorganisms CFU/g in feces compared with animals on sterile bedding (Turegeldiyeva et al. 2021). Microorganism products can cross the gut barrier and enter the circulatory system or vagal nerves (Lukiw et al. 2021; Zhao et al. 2021). Anaerobic Gram-negative bacteria in the gastrointestinal (GI) tract can produce the release of lipopolysaccharide (LPS), which is an immunogenic amphipathic glycoconjugate neurotoxic species that can induce systematic inflammation (Plociennikowska et al. 2015; Zhao et al. 2021).

APOE4 has been associated with oxidative damage and systemic inflammation, which are two mechanisms that are thought to accelerate AD. APOE4 is associated with an amplified inflammatory response to microbial products like LPS in both humans and mice (Grocott et al. 2001; Lynch et al. 2003; Vitek et al. 2009; Gale et al. 2014). Furthermore, peripheral inflammation is implicated in the exacerbation of microglial overactivation and the amplification of oxidative stress (OS) within the brain, both of which are pivotal factors in AD pathogenesis (Garcia-Dominguez et al. 2018; Pons and Rivest 2022). Variations in the microbiota, influenced by different housing conditions for experimental animals, may introduce variables that confound study outcomes, thereby hindering the translation of preclinical findings into clinically applicable therapies.

Preclinical studies utilizing APOE4 mouse models have enhanced our understanding of the pathogenesis of LOAD (Balu et al. 2019). To investigate the effect of housing conditions on key factors of LOAD pathogenesis, cohorts of hAPOE4/4 mice were initially bred in health status A conditions (barrier facility, helicobacter, and MNV negative, sterile food and housing). Upon reaching 22 months of age, half were moved to health status D housing conditions (non-barrier facility, helicobacter, and MNV negative, non-sterile food and housing). Two months later, blood, spleen, and brains were processed. Here, we report sex differences in brain cellular responses and immune status between sterile and non-sterile housing environments. The data presented in this study show the importance of reporting the housing health status in preclinical AD research utilizing mouse models to reduce variability and increase the interpretability of results. Reporting housing conditions may facilitate the potential translatability of findings, especially in the hAPOE4 knock-in animal model of LOAD.

## Materials and methods

### Animals

All animal studies and procedures were conducted using the National Institutes of Health guidelines for procedures on laboratory animals and were approved by the University of Arizona Institutional Care and Use Committee. Female and male humanized hAPOE4 targeted replacement B6(SJL)-APOE^tm1.1(APOE*4)Adiuj/J^ (Jackson Laboratories Stock No: 027894, humanized APOE4 KI) homozygous mice were obtained as breeding pairs and bred and aged at the University of Arizona. Female and male h*APOE4/4* mice were kept in health status A (helicobacter and MNV negative, sterile food, and housing) until 22 months of age. Thereafter, cohorts of mice were housed for 2 months in either a health status A or D (helicobacter and MNV negative, non-sterile food, and housing; *n* = 4/housing condition and gender). Mice were anesthetized with isoflurane, and blood was harvested via cardiac puncture. Mice were perfused with cold PBS, before the whole brain and spleen were collected.

### Brain, spleen, and whole blood cell isolation

Harvested whole brains were dissociated using the Miltenyi Biotec adult brain dissociation kit (Cat. #130-107-677), using the manufacturer’s protocol. Spleens were dissociated using the Spleen Dissociation Kit for mice (Miltenyi Biotec, Cat. #130-095-926). Blood samples were centrifuged, and plasma was collected and flash frozen at − 80 °C until used. Erythrocytes of the brain, spleen, and blood samples were removed using 1X Red Blood Cell Lysis Solution (Miltenyi Biotec, Cat. #130-094-183).

### Measurement of oxidative stress

MitoSOX™ (Invitrogen™, Cat. #M36008) staining was conducted to measure oxidative stress by first dissociating the whole brain into a single cell suspension followed by incubation with 5 μM MitoSOX™ for 15 min (37 °C, non-CO_2_ incubator) followed by PBS wash. After staining, the cells were resuspended, and DAPI (Miltenyi Biotec; Cat. #130-111-570) was added to exclude nonviable cells. Flow cytometry was used to analyze the results.

### Flow cytometry staining

Isolated cells from the whole brain, spleen, and blood were incubated with different combinations of antibodies (listed in Supplemental Table 1) for 25 min on ice. Cells were washed once with ice-cold PBS. Compensation controls were done for each experiment. After staining, the cells were resuspended, and DAPI was added to exclude nonviable cells. Fluorescence expression was determined using a MACSQuant10 flow cytometer (Miltenyi Biotec).

### Plasma protein measures

Cytokines and chemokines were measured using the V-PLEX Plus Pro-inflammatory Panel 1 Mouse Kit from MSD (Cat. # K15048D-1). Lipopolysaccharide-binding protein (LBP) concentrations were measured using a commercial ELISA kit (Cell Sciences Inc., Cat. #CKM043), samples were diluted 1:1000, and the assay was conducted according to kit protocol with a standard curve of 5–50 μg/mL.

### Statistical analysis

GraphPad Prism (GraphPad Software, San Diego, CA, USA) was used to analyze the data. To look for significant differences, means between more than two groups were analyzed using a two-way ANOVA, followed by post hoc Bonferroni testing. The level of statistical significance was set at 5%. All data are expressed as mean value ± standard deviation. Statistical tests for each experiment are provided in Supplemental Table 2.

## Results

To determine whether housing conditions influence brain OS and microglial activation, which are key factors driving LOAD pathogenesis, hAPOE 4/4 male (8) and female (8) mice were initially bred and housed in health status A conditions (helicobacter and MNV negative, sterile food, and housing (sterile F/H)). Upon reaching 22 months, half were moved to health status D housing conditions (helicobacter and MNV negative, non-sterile food, and housing (non-sterile F/H)) for 2 months.

### Sterile housing and food status reveal sex differences in oxidative levels in the brain in aged hAPOE mice

**Fig. 1:**
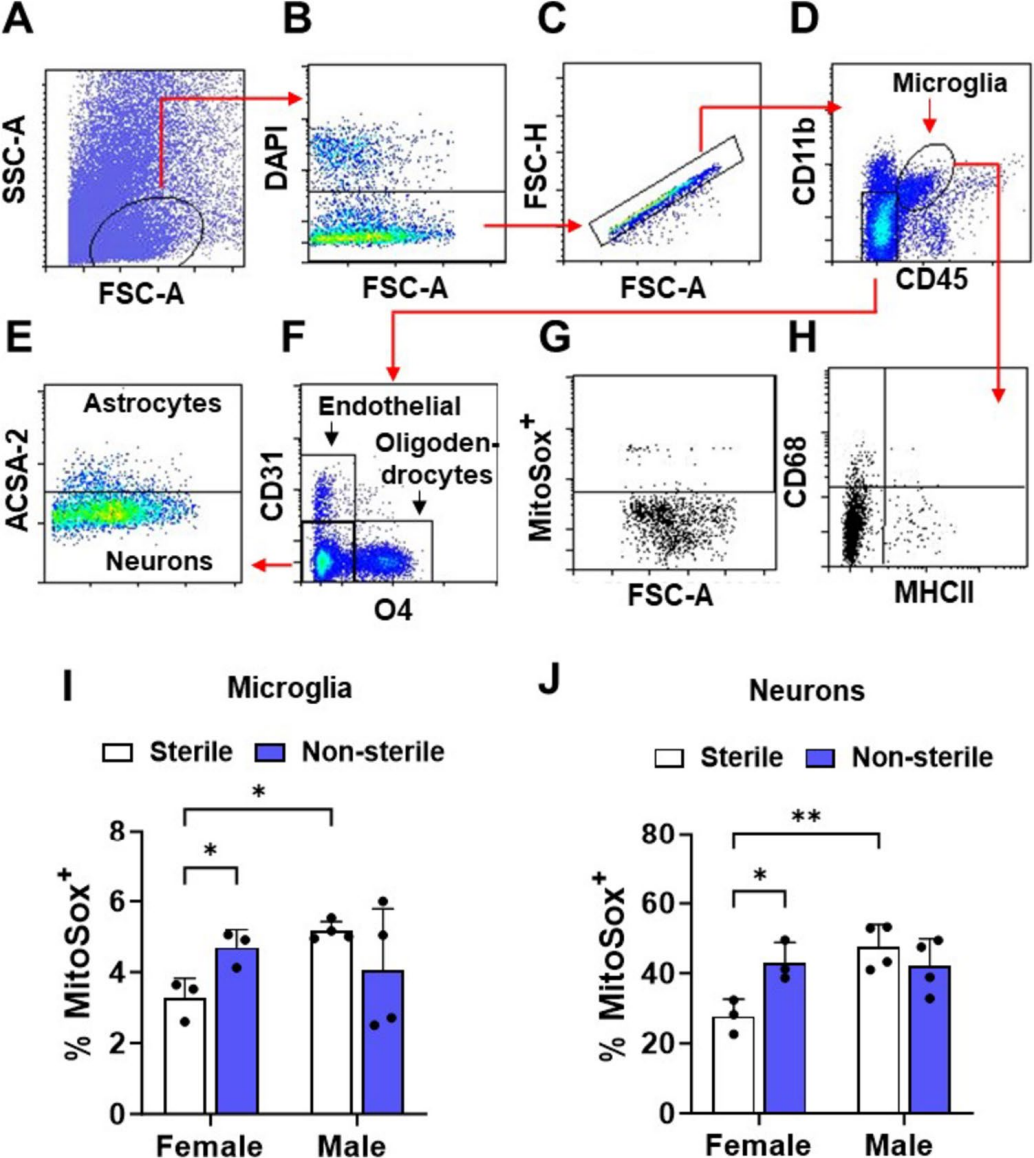
Oxidant production is increased in non-sterile conditions in hAPOE4 female, but not male, brain cells. Brain cells were dissociated from whole brain samples, stained for type-specific markers, and analyzed by flow cytometry. Panels (**A–H**) are representative graphs illustrating the brain cell gating strategy. **A** Brain cells were selected by their SSC and FSC characteristics. **B** Dead brain cells and **C** cell doublets were discriminated by their DAPI staining and FSC-A and FSC-H characteristics. Single brain cells were identified by gating expression of specific cell type markers: **D** Microglia expressing CD11b high and CD45 intermediate, **E** Astrocytes expressing ACSA-2^+^/CD45^−^/CD11b^−^, neurons expressing CD31^−^/O4^−^/ACSA-2^−^/CD45^−^/CD11b^−^, **F** oligodendrocyte expressing O4^+^/CD31^−^/ACSA-2^−^/CD45^−^/CD11b^−^ and endothelial cells by expression of CD31^+^/O4^−^/ACSA-2^−^/CD45^−^/CD11b^−^. **G** Each brain cell type was used for gating mitochondrial oxidative stress (MitoSox^+^). **H** Microglial cells were gated for expression of MHC-II and CD68. Quantification of mitochondrial oxidative stress in microglia (**I**) and neurons (**J**). Data are represented as mean ± SD. *p* values were calculated using two-way ANOVA (Bonferroni adjusted *p*-values are shown in Supplementary Table 2; *p ≤ 0.05, **p ≤ 0.01)

Brains were collected and processed to generate single brain cell suspensions of each group. Brain cell suspensions were labeled with MitoSox, which generates a red fluorescent product when reacting with oxidants (Kalyanaraman 2020). First, brain cells were gated from total events (Fig. 1A), dead cells (DAPI + ; Fig. 1B), singlets (Fig. 1C), and expression of cell-type-specific markers: microglia (αMβ2 integrin high expression (CD11b^high^) and receptor-linked protein tyrosine phosphatase intermediate expression (CD45^intermediate^), which are surface markers to evaluate the microglia (Milner et al. 2022; Fig. 1D), astrocytes (astrocyte cell surface antigen-2 (ACSA-2^+^); Fig. 1E), neurons (CD11b^−^/CD45^−^/CD31^−^/O4^−^/ACSA-2^−^; Fig. 1E), oligodendrocytes (HSO3-3-galactosylceramide the major glycosphingolipid components of oligodendrocytes (O4^+^); Fig. 1F) and endothelial cells (Platelet/endothelial cell adhesion molecule-1 (CD31^+^), Fig. 1F). Subsequently, each brain cell type was MitoSox^+^ gated (Fig. 1G). Compared to sterile F/H, non-sterile F/H induced a significant increase in the percent of MitoSox^+^ microglia (*p* = 0.0144) and neurons (*p* = 0.0146) in hAPOE4 females and not in males. Endothelial cells, oligodendrocytes, and astrocytes exhibited no change in the percentage of MitoSox^+^ cells between sterile and non-sterile F/H in either hAPOE4 females or males (Supplementary Fig. 1A–C). In sterile F/H, hAPOE4 males exhibited a greater percent of MitoSox labeling in microglia and neurons relative to hAPOE females (Fig. 1I, J). Nevertheless, these sex differences were not observed in non-sterile F/H, perhaps due to the response of the female mice to the non-sterile environment (Fig. 1I, J). These results suggest that housing status impacts the production of oxygen radicals and OS in the brain. These results also suggest that environmental status may be important in studies looking at sex differences, and especially in aged hAPOE4 mice.

### Aged female and male hAPOE mice exhibit different microglial activation responses to housing conditions

**Fig. 2:**
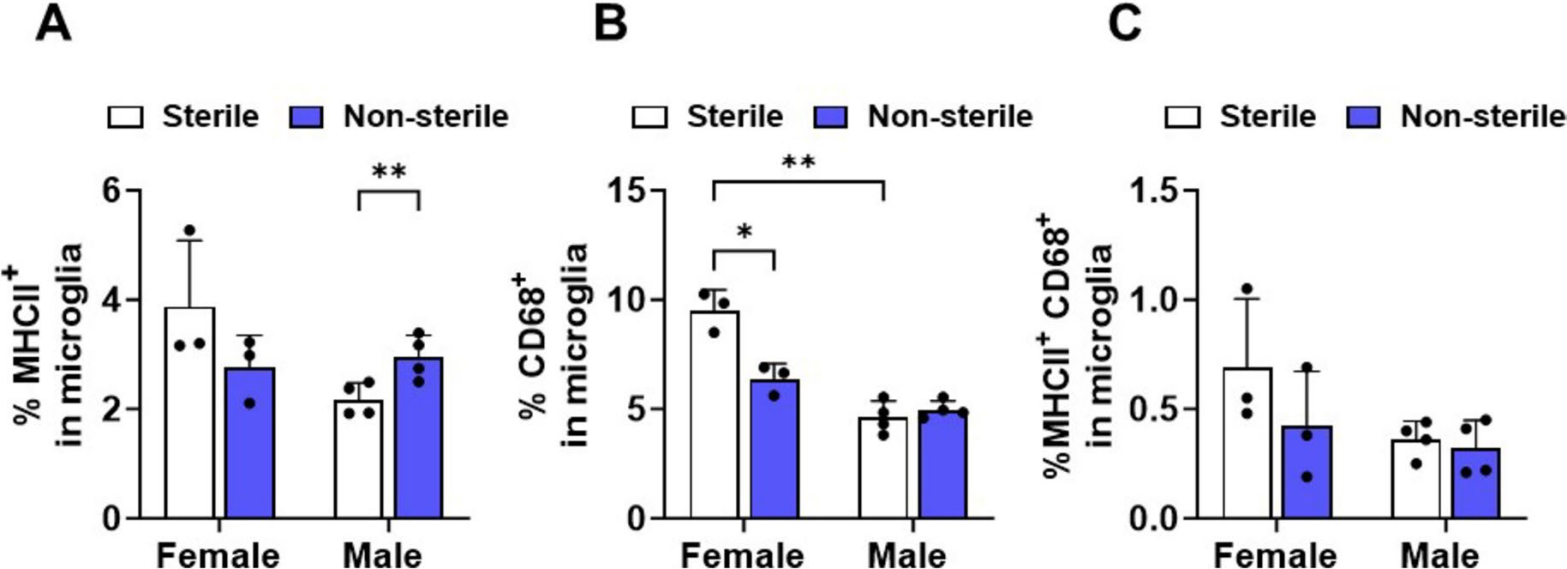
Microglia activation is affected by housing and food status. Quantification of expression of microglial activation markers **A** MHCII^+^, **B** CD68^+^, and **C** MHCII^+^ CD68^+^. Data are represented as mean ± SD. *p* values calculated using two-way ANOVA (Bonferroni adjusted *p*-values are shown in Supplementary Table 2; *p ≤ 0.05, **p ≤ 0.01, and ***p ≤ 0.001)

Microglia, as the primary immune cells of the central nervous system, play a pivotal role in neuroinflammatory responses and are increasingly recognized for their sensitivity to peripheral changes, including those stemming from the gut (Hoogland et al. 2015; VanItallie 2017; Kesika et al. 2021; Leng and Edison 2021). Microglia activation was investigated by determining the expression of MHCII and CD68 microglial reactivity indicators (Fig. 1H). CD68 is a transmembrane glycoprotein that signifies phagocytic activity (Rabinowitz and Gordon 1991). MHCII expression points to a more immune-activated microglial phenotype with a propensity for proliferation (Styren et al. 1990). In hAPOE4 females, no significant change in the expression of MHCII (Fig. 2A) across housing conditions was evident. In contrast, male hAPOE4 mice exhibited a greater percentage of MHCII-positive microglia (*p* = 0.0039) housed in non-sterile F/H compared to sterile F/H. hAPOE4 females in non-sterile F/H exhibited a significant decrease in the percentage of phagocytic marker CD68^+^ microglia compared with those under sterile F/H (*p* = 0.0455). No difference in the percent of CD68^+^ was observed in hAPOE4 males across housing conditions (Fig. 2B). Sterile and non-sterile did not affect the co-expression of MHCII and CD68 in either aged hAPOE4 females or males (Fig. 2C). Sex differences were observed under the sterile F/H condition. Microglia from hAPOE4 females housed under sterile conditions exhibited greater expression of phagocytic microglial indicator CD68^+^ (*p* = 0.0035) relative to hAPOE4 males (Fig. 2B). In contrast, no sex differences were observed under the non-sterile F/H condition.

### Sex difference in the infiltration of CD4^+^ T cells into the brain was increased in hAPOE4 males moved in non-sterile food and housing

**Fig. 3:**
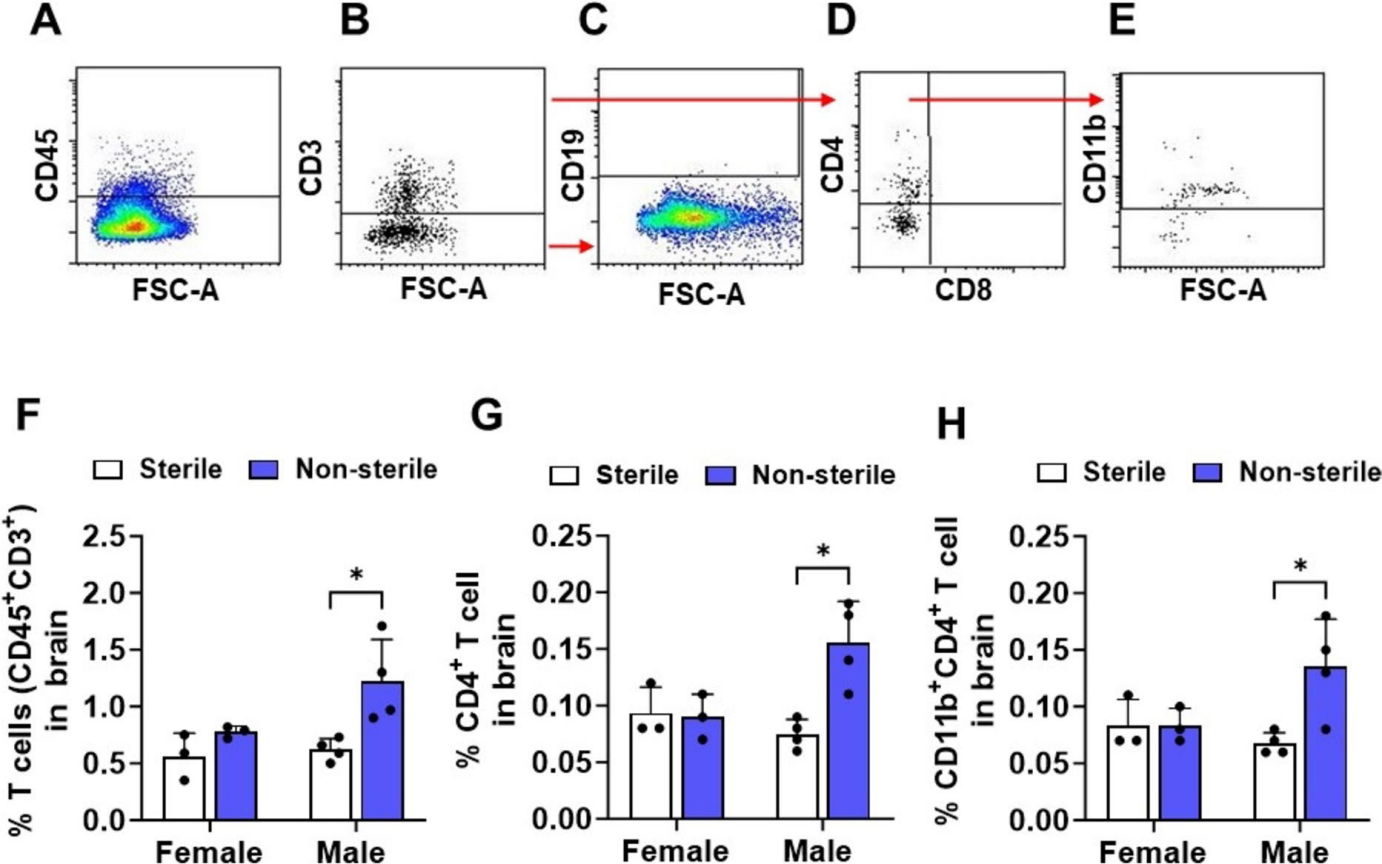
Non-sterile environments increase the infiltration of T cells into the male, but not female brain. Male and female APOE4 mouse brains were dissociated and assessed using a single-cell suspension. Brain cells were stained for immune cell markers and analyzed by flow cytometry. **A** After discriminating dead cells and doublets, immune cells were gated by expressing CD45^+^. Immune cells were classified as **B** T cells (CD45^+^ CD3^+^), **C** B cells (CD19^+^/CD45^+^/CD3^−^) and **D** helper T cells (CD4^+^/CD3^+^/CD45^+^) and cytotoxic T cells (CD8^+^/CD3^+^/CD45^+^). **E** Helper T cells were gated for expression of CD11b. Quantification of **F** total T cells, **G** helper T cells and **H** CD11b^+^ helper T cells in the brain. Data are represented as mean ± SD. *p* values were calculated using two-way ANOVA (Bonferroni adjusted *p*-values are shown in Supplementary Table 2; *p ≤ 0.05, and **p ≤ 0.01)

Activated microglia promote cytokine production that can induce immune cell infiltration into the brain (Leng and Edison 2021). MHCII expression points to a more immune-activated microglial phenotype with a propensity for proliferation and plays a crucial role in mobilizing immune cells to an inflammatory response under pathological conditions (Styren et al. 1990). Our data above showed that housing conditions affect microglia activation. Therefore, to investigate whether housing status affects the infiltration of immune cells into the brain, isolated brain cells of hAPOE4 females and males were labeled with immune cell-type-specific markers and sample assessment gated for the leukocyte common antigen CD45 (Fig. 3A). Leukocytes were classified as T cells (CD45^+^/CD3^+^, Fig. 3B), helper T cells (CD45^+^/CD3^+^/CD4^+^, Fig. 3D), cytotoxic T cells (CD45^+^/CD3^+^/CD8^+^, Fig. 3D), and B cells (CD45^+^/CD19^+^, Fig. 3C). In the hAPOE4 male brains, non-sterile F/H was associated with a nearly twofold increase in the percent of the T cells in the brain compared to non-sterile F/H (1.2 vs. 0.59%, *p* = 0.0248, Fig. 3F). No difference was detected in the hAPOE4 female brain. Under non-sterile F/H, CD4^+^ T cells increased in the brains of hAPOE4 males compared with sterile F/H mice (0.14 vs. 0.07%, *p* = 0.0438, Fig. 3G). Interestingly, migratory CD4^+^ T cells in the male brain also expressed CD11b^+^ (Fig. 3H).

### Non-sterile food and housing increased the expression of CD11b^+^ in circulatory CD4^+^ T cells and B cells in hAPOE4 males, but not in females

**Fig. 4:**
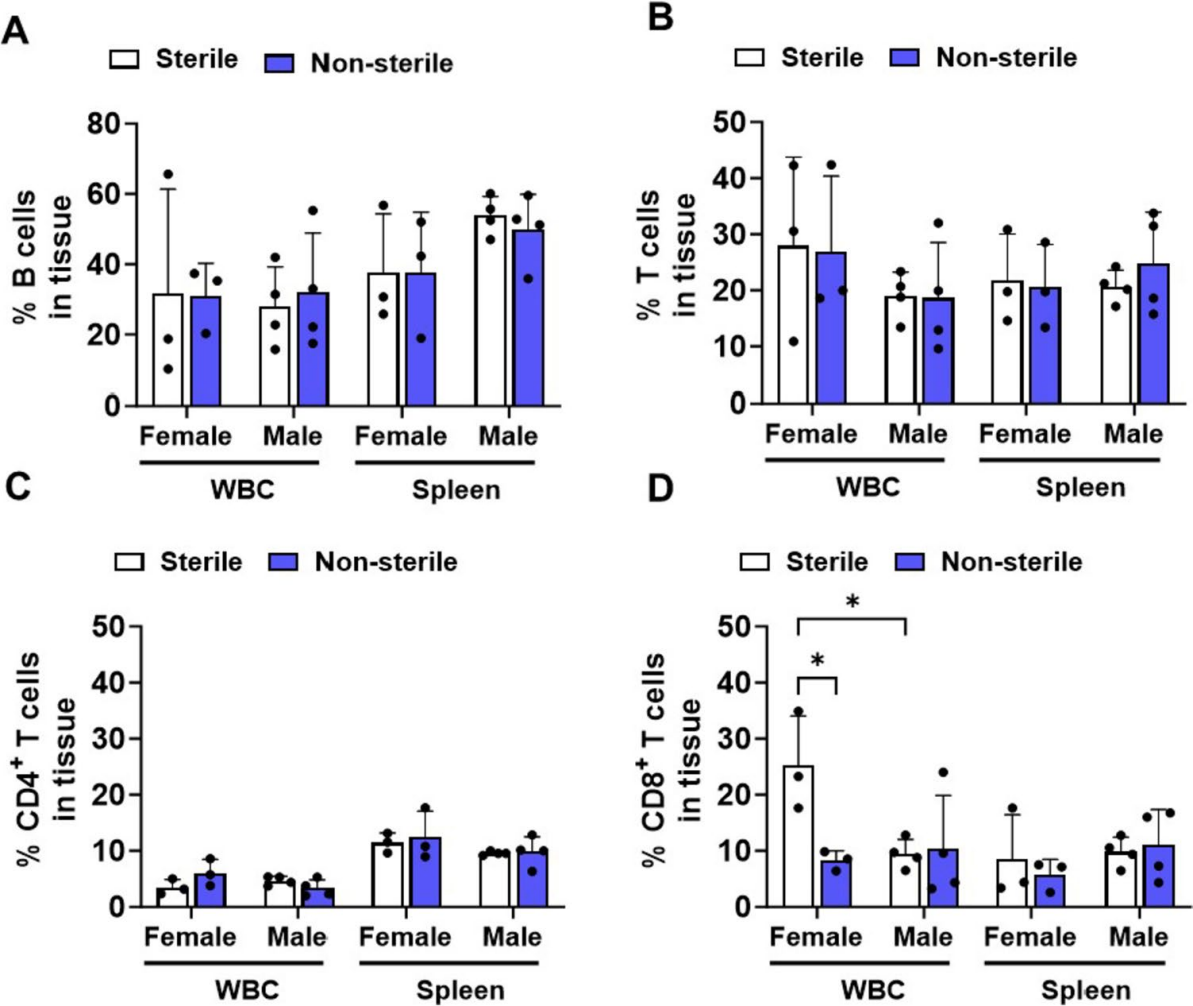
Peripheral adaptive immune cells are affected by sterile and non-sterile food and housing conditions. White blood cells (WBC) and splenic cells were isolated and stained immune cell markers and analyzed by flow cytometry. **A** Quantification of % B cells (CD19^+^/CD45^+^/CD3^−^), **B** T cell (CD3^+^/CD45^+^), **C** helper T cell (CD4^+^/CD3^+^/CD45^+^) and **D** cytotoxic T cells (CD8^+^/CD3^+^/CD45^+^) in spleen and WBC. Data are represented as mean ± SD. *p* values were calculated using two-way ANOVA (Bonferroni adjusted *p*-values are shown in Supplementary Table 2; *p ≤ 0.05, **p ≤ 0.01, ***p ≤ 0.001)

Since non-sterile F/H increased the migration of CD11b^+^/CD4^+^ T cells into the brain, we investigated whether housing conditions altered peripheral adaptive immune cells. To this end, white blood cells (WBC) and spleen cells from hAPOE4 females and males were labeled with immune cell-type-specific markers, CD11b inflammatory, and CD69 T cell activation markers followed by flow cytometry analyses. Labeled cells were gated as described in Fig. 3A–E. The percentage of total B cells (Fig. 4A), total T cells (Fig. 4B), and CD4^+^ T cell subset (Fig. 4C) were not affected by different housing conditions or by chromosomal sex in either blood or spleen. However, the percentage of CD8^+^ T cells in the blood, not in the spleen, was affected by environmental housing and food status. The percent of CD8^+^ T cells in the blood of hAPOE4 females in sterile F/H was significantly higher than in non-sterile F/H (*p* = 0.0147) and also compared to hAPOE4 males (*p* = 0.01) in the same housing conditions (Fig. 4D). The sex difference in the percent of CD8^+^ T cells was not apparent under non-sterile F/H.

**Fig. 5:**
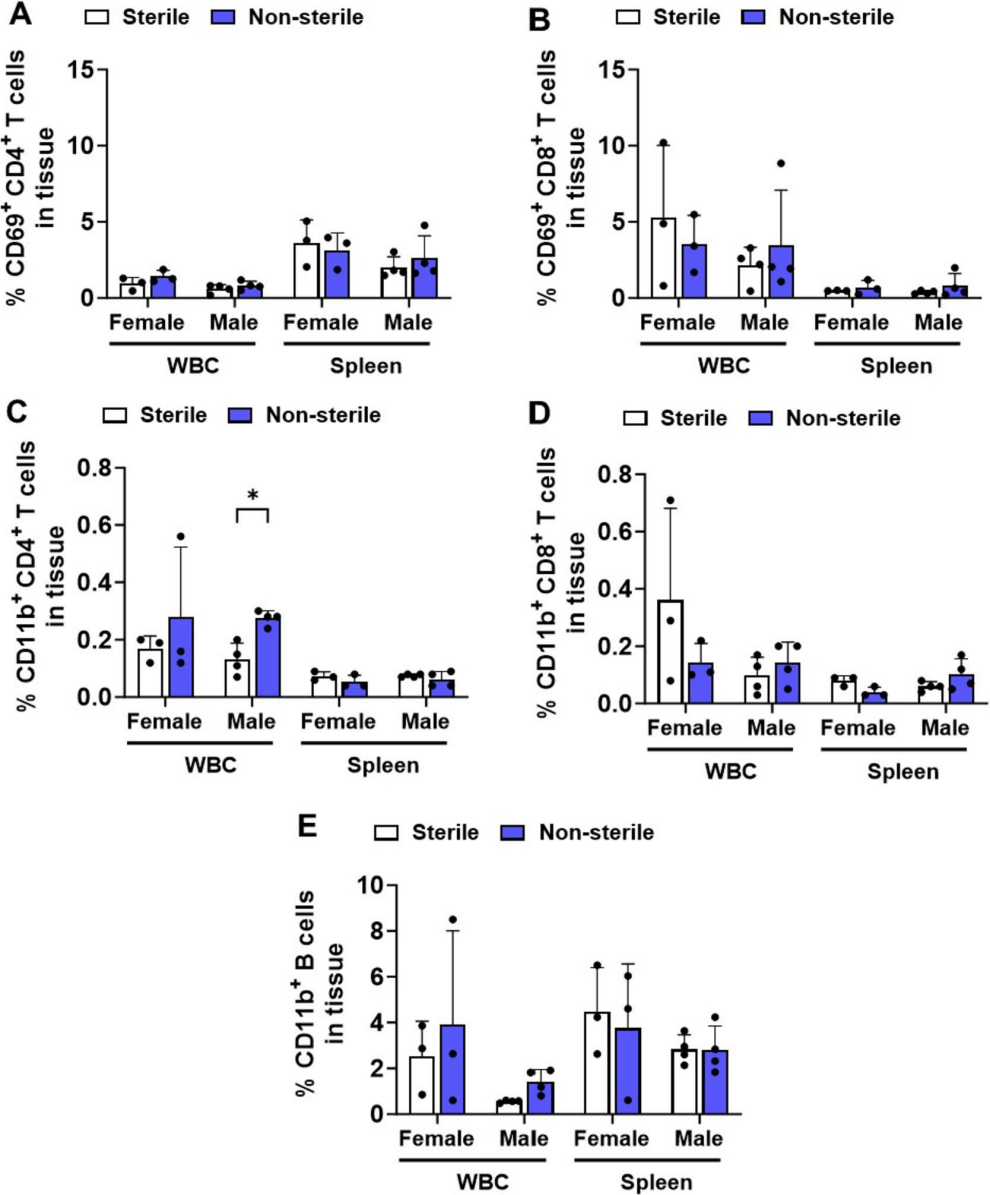
Sterile and non-sterile food and housing effects on the expression of T cell activation and inflammatory markers. Splenic cells and WBCs were stained for B cell and T cell markers (CD3^+^, CD4^+^, CD8^+^), the T cell activation marker CD69^+^, and inflammation marker CD11b^+^ and analyzed by flow cytometry. CD69^+^ was measured in both **A** CD4^+^ and **B** CD8^+^ T cells in the blood and in the spleen (**C**, **D**). CD11b^+^ was measured in **C** CD4^+^ and **D** CD8^+^ T cells and **E** B cells in the blood and in the spleen. Data are represented as mean ± SD. *p* values were calculated using two-way ANOVA (Bonferroni adjusted *p*-values are shown in Supplementary Table 2; *p ≤ 0.05 and **p ≤ 0.01)

We further sought to evaluate T cell activation through the expression of CD69 by T cells. T cells express CD69 rapidly upon stimulation of the T cell receptor (TCR) (Testi et al. 1989; Yamashita et al. 1993), and IL-2 is produced by activated T cells in response to antigen stimulation (Malek 2008). The percentage of CD4^+^ and CD8^+^ T cells expressing CD69 in WBC or spleen of hAPOE4 females and males were similar across sterile and non-sterile F/H conditions (Fig. 5A, B). IL-2 levels in the serum of hAPOE4 females and males were also similar between sterile and non-sterile F/H (Supplementary Fig. 2). These results indicate that the environmental housing and food statuses tested in this study had not affected T cell activation.

In contrast, white blood cells (WBC) of hAPOE4 males housed in non-sterile conditions exhibited a significant increase in the percentage of CD11b^+^ CD4^+^ T cells (*p* = 0.0276) compared to hAPOE4 males housed in sterile F/H (Fig. 5C). No significant changes were observed in the percentage of CD11b^+^ CD8^+^ T cells and CD11b^+^ B cells (Fig. 5D, E). hAPOE4 females exhibited no significant differences in the percentage of CD11b^+^ adaptive immune cells between sterile and non-sterile housing or compared to males in WBC or spleen (Fig. 5C–E). These data suggest that aged hAPOE4 males are more susceptible to an inflammatory response in non-sterile F/H than aged hAPOE4 females.

### Non-sterile food and housing increase the lipopolysaccharide levels in blood in aged hAPOE4 males

APOE4 has been associated with a greater inflammatory response to microbiome products. Bacterial products, such as LPS, release signaling to promote neurotoxicity (Lukiw et al. 2021; Zhao et al. 2021) by increasing microglia activation and inducing MHCII expression (Casals et al. 2007; Hoogland et al. 2015; Sharma and Nehru 2015; Zhao et al. 2022). LPS has also been reported to increase the expression of CD11b (Lynn et al. 1991; Zhou et al. 2005; Kotsougiani et al. 2010). Because the data above indicated that housing and food status promoted changes in oxygen radical levels, microglia activation, and expression of CD11b in CD4^+^ T cells, we investigated if these responses were related to changes in the LPS levels in mouse circulation. Direct measures of LPS in plasma present technical difficulties because of  its lack of sensitivity and the requirement to collect the samples under LPS-free conditions. However, circulating LPS-binding protein (LBP), a plasma protein that binds to LPS, has been reported to correlate highly with LPS levels (Lepper et al. 2007; Moreno-Navarrete et al. 2012).

**Fig. 6:**
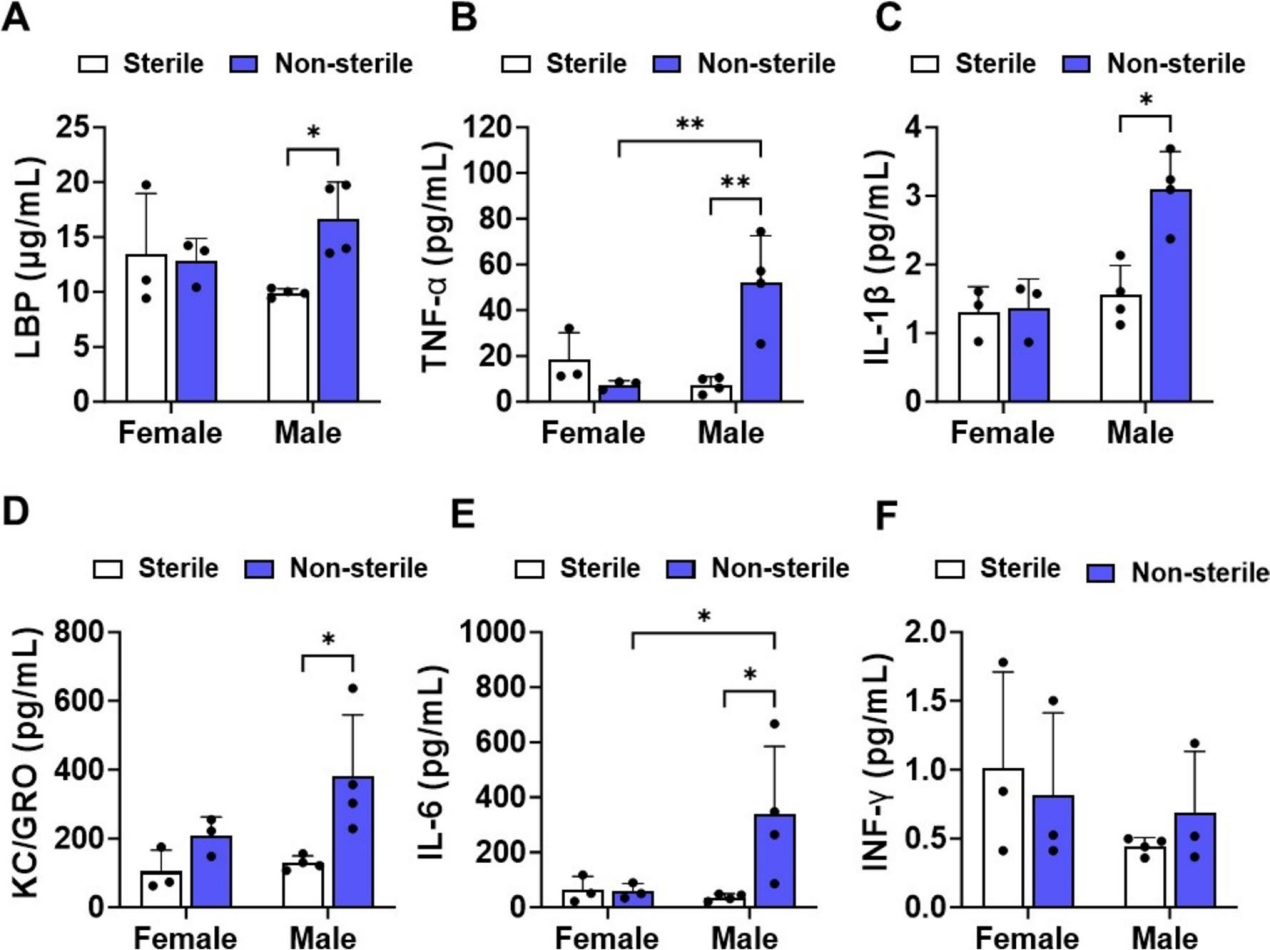
Non-sterile food and housing increase the levels of LPS-binding protein and inflammatory cytokines in the plasma of APOE4 males. The concentration of circulating **A** LPS-binding protein (LBP), **B** TNF-α, **C** IL-1β, **D** KC/GRO, **E** IL-6, and **F** INF-γ- were measured from plasma collected at necropsy. Data are represented as mean ± SD. *p* values were calculated using two-way ANOVA (Bonferroni adjusted *p*-values are shown in Supplementary Table 2; *p ≤ 0.05 and **p ≤ 0.01)

In the non-sterile F/H, hAPOE4 males, not females, exhibited higher LBP levels in plasma than males housed in sterile F/H (Fig. 6A). LPS causes inflammatory activation mainly through binding Toll-like receptor 4 (TLR4), which activates nuclear factor κB (NFκB), thereby promoting the transcription of multiple inflammatory factors, including IL-6, IL-1β, TNF-α, and KC/GRO. LBP cooperates with CD14 to facilitate the transfer of LPS to TLR4. Accordingly, an increase of LPS and LBP in plasma can be expected to enhance the levels of inflammatory cytokines. Consistent with this pathway, hAPOE4 males housed in non-sterile F/H, but not females, exhibited higher levels of IL-6, IL-1β TNF-α, and KC/GRO in plasma than hAPOE4 males maintained in sterile F/H (Fig. 6B–E). However, the level of INF-γ, whose production is not controlled by LPS, was not affected by housing and food status changes or sex (Fig. 6F).

## Discussion

APOE4 is the greatest genetic risk factor for developing AD (Michaelson 2014; Safieh et al. 2019; Sienski et al. 2021). hAPOE4 transgenic (Tg) mice have been developed to model the phenotypic pathology and disease progression, including amyloid plaques (amyloid-β peptide/ Aβ), intracellular neurofibrillary tangles (tau; NFT), neuroinflammation, neuritic dystrophy, frank neuronal loss, and learning/memory deficits (Balu et al. 2019). However, APOE4 mice showed a greater inflammatory response to microbiome products than mice not expressing an APOE4 allele (Vitek et al. 2009; Gale et al. 2014). The mechanism whereby APOE4 may promote this response remains unknown, but APOE4 pro-inflammatory response has been associated with the impact of APOE4 in modulating the gut microbiome and innate immune response (Vitek et al. 2009; Zhao et al. 2014; Tran et al. 2019). Therefore, the interaction of hAPOE4 with environmental microorganisms may have a significant impact on the experimental results.

Research institutions use different rodent housing systems to limit microbial contamination. The University of Arizona vivarium uses barrier animal facilities to maintain an A status (helicobacter and MNV negative, sterile food, and housing), and non-barrier animal facilities which keep a D status (helicobacter and MNV negative, non-sterile food, and housing). The goal of this study was to understand potential sources of variation in data from aged hAPOE4 animals housed in barrier vs. non-barrier facilities. The findings of this study indicate that exposure of two modifiable factors, sterility of housing and food, can impact microglial function, immune activation, and peripheral T cell infiltration into the brain of aged hAPOE4 mice after only two months. It is important to acknowledge that the results of this study may primarily pertain to older mice (22–24 months), equivalent to humans between 56 and 69 years old (Dutta and Sengupta 2016). This age range is known to have the highest risk of developing Alzheimer's disease (Guerreiro and Bras 2015).

We observed that non-sterile F/H hAPOE4 females showed higher oxidative stress (OS) levels than in sterile F/H without accompanying changes in immune-activated microglial phenotype (MHCII) (Figs. 1I, 2A). Conversely, non-sterile F/H males showed a higher immune-activated microglial phenotype than sterile F/H without changes in OS (Figs. 1I, 2A). These findings could seem to be inconsistent. However, microglial activation can be triggered by various exogenous and endogenous stimuli such as bacterial LPS, pathogen-associated molecular patterns (PAMPs), pathogen genetic material, danger/damage-associated molecular patterns (DAMPs), and protein aggregates. Depending on the stimulus, activated microglia can perform functions such as phagocytosis, cytokine and chemokine secretion, antigen presentation, and reactive oxygen species (ROS) production (Venneti et al. 2009; Streit 2010; Leng and Edison 2021; Wang et al. 2023). It is crucial to understand that although activated microglia are capable of producing ROS, they do not invariably do so since ROS production is contingent upon the context. ROS generation in cells arises through multiple mechanisms, often by-products of cellular metabolism or pathways such as glucose metabolism, mitochondrial electron transport chain (ETC), xanthine oxidase pathway, arachidonic acid metabolism, and auto-oxidation pathways. ROS plays pivotal roles in cell signaling, and cells harbor antioxidant mechanisms to detoxify ROS and ensure a balanced state. OS is a phenomenon caused by an imbalance between the production and accumulation of ROS in cells and tissues and the ability of a biological system to detoxify these reactive products (Pizzino et al. 2017). APOE is known to have antioxidant properties, with ApoE4 being the least effective in this role (Brown et al. 2002; Colton et al. 2002; Butterfield and Mattson 2020). OS levels in hAPOE4 microglia are not a definitive marker of their activation state. Therefore, altered OS levels in microglia of hAPOE4 may not necessarily represent microglia activation phenotypes.

**Fig. 7:**
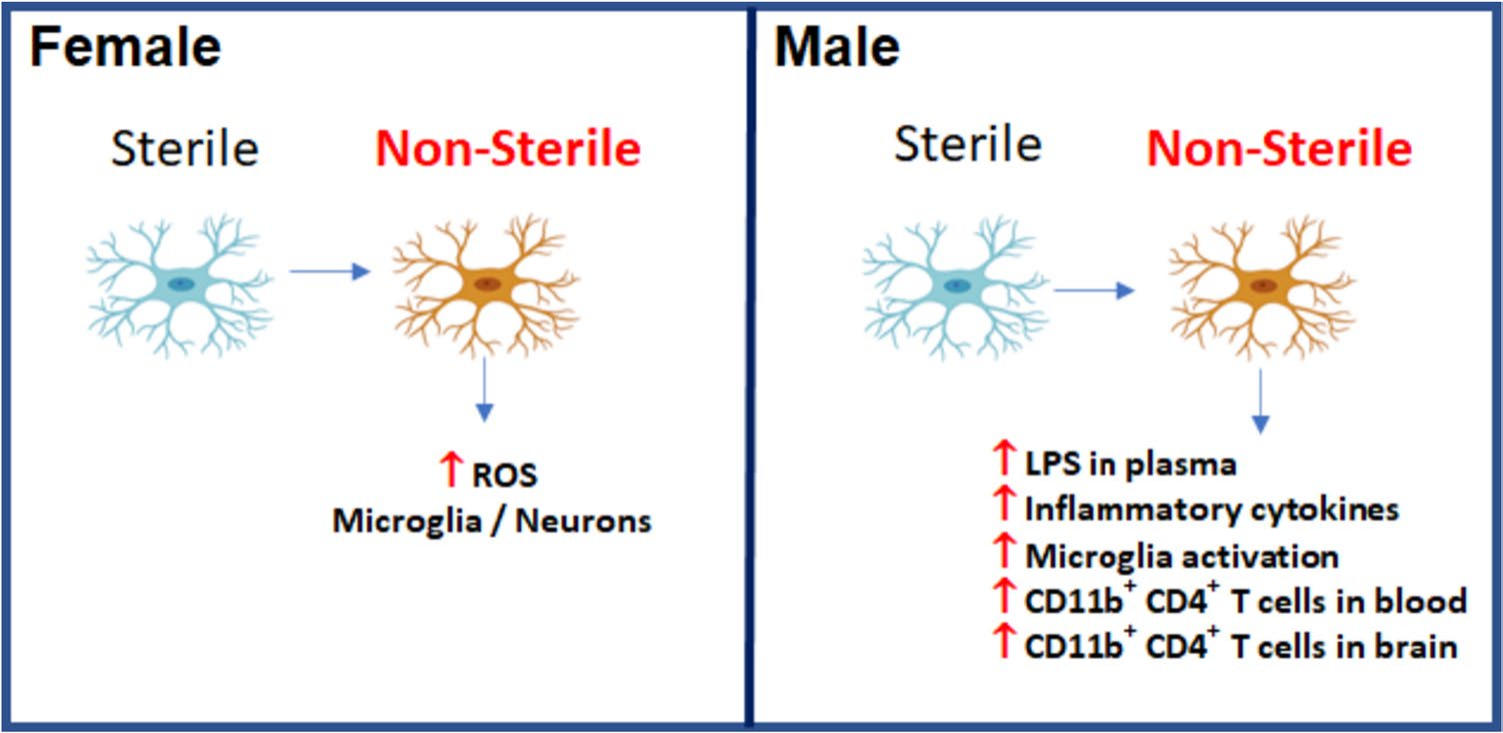
Aged hAPOE4 males are more susceptible to housing and food status than aged hAPOE4 females. hAPOE4 females and males responded differently to sterile and non-sterile housing and food status. Female hAPOE4 mice moved to non-sterile conditions increase in MitoSox + microglia and neurons and did not exhibit an inflammatory response to non-sterile food and housing conditions. However, male hAPOE4 mice exposed to non-sterile food and housing exhibited an increase in the levels of LPS and inflammatory cytokines in the circulatory system, activated microglia, infiltration of T cells into the brain compared to those in sterile food and housing conditions

Microglia play a regulatory role in the central nervous system. After an insult or injury, microglia become activated, exhibiting increased motility and production of cytokines (Liu et al. 1998; Carbonell et al. 2005). Microglial activation can evoke processes that lead to T cell infiltration into the brain (Gemechu and Bentivoglio 2012; Gate et al. 2020; Jin et al. 2021). Our data indicate that aged hAPOE4 males exposed to non-sterile food and housing exhibited an increase in activated microglia, infiltration of T cells into the brain, and levels of inflammatory cytokines in the circulatory system compared to those in sterile food and housing conditions (Fig. 7). Inflammatory signals, particular pro-inflammatory cytokines induce the expression of molecules necessary to immune cell infiltration into the CNS (Murphy et al. 2002; Sporici and Issekutz 2010). CD11b is an adhesion protein and is considered an inflammatory marker. CD11b has been reported to be a homing receptor of T lymphocytes for nonlymphoid tissue such as an infected or inflamed site (Nielsen et al. 1994; Bullard et al. 2005; Mindur et al. 2014). Aged hAPOE4 males housed in non-sterile conditions showed an increase in T cells, particularly inflammatory CD11b^+^CD4^+^ T cells in the brain. However, B and CD8^+^ T cells were not detected in the brain under any experimental conditions tested in this study. Moreover, we observed that the corresponding phenotype of peripheral cells is linked with the infiltration of adaptive immune cells into the brain. In males moved to non-sterile housing conditions, there was an increase in migratory, inflammatory T cells (CD4^+^CD11b^+^ T cells) in the blood. A study by Bullard showed that lack of CD11b prevents T cell infiltration into the brain (Bullard et al. 2005). These findings suggest that housing conditions may influence the migratory properties of adaptive immune cells.

One means by which housing and food status may affect immune status is through changes in gut integrity and microbiome, as supported by previous research (Bidot et al. 2018; Ericsson et al. 2018; Korte et al. 2018). Although our study did not analyze fecal samples to identify specific microbiome changes associated with different housing and food statuses, our results suggest that exposure to gram-negative bacteria might be responsible for the observed immune changes. In this context, our study found that aged hAPOE4 male mice in non-sterile environments exhibited increased plasma levels of LBP compared with their counterparts in sterile conditions. The correlation between LBP and levels of LPS, a component of Gram-negative bacteria (Lepper et al. 2007; Moreno-Navarrete et al. 2012, 2017), implies that elevated LBP can be a marker for LPS in plasma, indicative of Gram-negative bacterial contamination and the potential crossing of LPS across the gut barrier.

The ability of gut bacterial products to cross the gut barrier, enter the systemic circulation, and impact immune and neurological health is well documented (Bryant et al. 2010; Lukiw et al. 2021; Zhao et al. 2021). LPS initiates an inflammatory response via activation of toll-like receptor 4 (TLR4) (Bryant et al. 2010). LBP binds to LPS and transfers it to CD14 on cell membranes, which then presents LPS to TLR4 (Kim and Kim 2017). This interaction activates the NF-κB signaling pathway, leading to the transcription of inflammatory genes, which include cytokines like TNFα, IL-6, and pro-IL-1β (Morris et al. 2014). In our findings, mice housed in non-sterile conditions showed increased levels of these pro-inflammatory cytokines in their plasma. LPS can also promote neurotoxicity by activating microglia, potentially causing neuroinflammation (Huebbe et al. 2015; Sharma and Nehru 2015). Therefore, our observations indicate that non-sterile housing in facilities with a D health status likely promotes contamination by gram-negative bacteria, which in turn may elevate LPS levels and trigger systemic inflammation. Such systemic inflammation could lead to a neuroinflammatory response, disrupting neuronal and glial function.

Aged hAPOE4 females responded differently to the housing and food status tested. They did not exhibit an inflammatory response to non-sterile conditions, including no increase in microglial activation, T cell infiltration into the brain, or inflammatory cytokines. The only change observed in the aged female hAPOE4 mice moved to non-sterile conditions was an increase in MitoSox + microglia and neurons (Fig. 7). It is noteworthy that sex differences have been reported in ApoE4 carriers in relation to metabolic alteration. Additionally, APOE4 has associations with impaired glucose metabolism (Huebbe et al. 2015; Farmer et al. 2021; Zhao et al. 2021). Studies like Mattar et al. (2022) have reported that hAPOE4 female mice are more susceptible to metabolic disturbances than males (Mattar et al. 2022). Therefore, we speculate that our findings showing different levels of OS in the microglia and neurons of females and males hAPOE4 mice in different vivarium housing conditions might be attributed to metabolic differences influenced by sex.

The differential impact of AD on cognitive decline and neuropathology between sexes is well-documented. Females exhibit more severe cognitive impairment and neuropathological manifestations than males, as shown in various studies (Alzheimer’s Association Report 2021a, b; Zhang et al. 2021; Tzioras et al. 2023). It has been observed that women who are APOE4 carriers experience faster cognitive decline and amyloid-beta (Aβ) accumulation compared to men with the same genetic makeup (Corder et al. 2004). This sex–APOE4 genotype interaction significantly influences a range of AD-related outcomes (Christensen et al. 2010; Altmann et al. 2014; Kanekiyo et al. 2014; Tao et al. 2018; Duarte-Guterman et al. 2020; Farmer et al. 2021; Polsinelli et al. 2023). For instance, males with APOE4 tend to have more cerebral microbleeds, whereas females with the same allele predominantly develop plaques and tangles (Cacciottolo et al. 2016). Moreover, cognitive decline and amyloid-beta (Aβ) accumulation progress faster in female APOE4 carriers than in males (Corder et al. 2004). The mechanisms of how APOE4 confers a stratified risk for women are not fully understood, but evidence suggests links to sex hormones like estrogen, and differences in how APOE4 affects metabolism and gut microbiome in a sex-dependent manner (Haro et al. 2016; Maldonado Weng et al. 2019; Arnold et al. 2020; Gamache et al. 2020; Holingue et al. 2020; Mattar et al. 2022). Our study contributes to this discussion by showing that aged male hAPOE4 mice are particularly sensitive to non-sterile housing and food conditions, responding with an increased inflammatory reaction compared to females. However, female hAPOE4 mice in non-sterile F/H conditions had greater OS levels than those in sterile conditions, without inflammatory response changes (Fig. 7). These findings suggest a heightened susceptibility in aged hAPOE4 males to environmental factors that can alter immune responses by modifying gut integrity and microbiome composition. Distinct gut microbiome profiles have been reported in male and female hAPOE4 mice (Zajac et al. 2022), reinforcing the notion of male-specific vulnerability to environmental influences on gut microbiota. Diet and nutrition further modulate the gut microbiota's composition (Mattar et al. 2022; Chang et al. 2023). In this vein, the work of Mattar et al. highlights that male hAPOE4 mice on a high-fat western diet demonstrated liver dysfunction and heightened levels of brain inflammatory cytokines, but female hAPOE4 mice on the same diet did not show notable changes in liver weight or brain inflammatory cytokines expression. In conjunction with ours, these results point to a potentially more pronounced sex difference and shift toward inflammation in the gut microbiome of hAPOE4 male mice.

Future research should explore whether the oxidation state, microglial activation, and immune responses observed in aged hAPOE4 mice under various housing conditions have correlations with cognitive outcomes and whether these outcomes display sex-specific differences. The immune system's response to aging differs between the sexes: females show increased genomic activity in adaptive immune cells, whereas males have more innate and pro-inflammatory activity and reduced adaptive activity (Marquez et al. 2020). Furthermore, sex differences in systemic inflammation have been linked to heightened AD pathology in humans (Walker et al. 2018). The APOE4 allele is shown to influence cytokine levels differently across sexes, with females having lower levels than males (Duarte-Guterman et al. 2020). The Atherosclerosis Risk in Communities (ARIC) study explored the relationship between midlife systemic inflammation and cognitive decline over a span of 20 years, concluding that higher levels of circulating inflammatory markers in midlife are associated with subsequent cognitive decline. However, the study did not demonstrate any modification of this effect by sex or APOE4 status (Walker et al. 2018). Given that cognitive decline is strongly associated with sex and APOE4 status (Duarte-Guterman et al. 2020; Polsinelli et al. 2023), these findings suggest that systemic inflammation may indicate neurodegenerative disease or injury to neurons or glial cells, but it may not necessarily be a direct marker for cognitive decline. This is further supported by the finding that female hAPOE4 mice on a high-fat Western diet for 9 months showed spatial learning and memory deficits without the increased brain inflammation markers seen in male mice (Mattar et al. 2022). Nonetheless, OS and free radical damage have been implicated in the cognitive impairment seen in AD patients (Lovell and Markesbery 2007; Padurariu et al. 2010; Chico et al. 2013). Our study showed that female hAPOE4 mice in non-sterile F/H conditions had greater OS levels in microglia and neurons than those in sterile conditions, a change not seen in the inflammatory responses of males. From these data, we hypothesize that aged female hAPOE4 mice might experience greater cognitive decline in non-sterile F/H conditions compared to females in sterile F/H and males in non-sterile F/H.

In summary, preclinical research using APOE4 mouse models has significantly enriched our understanding of LOAD pathogenesis (Balu et al. 2019). Our findings indicate that the environmental living conditions of aged hAPOE4 mice can impact microglial activation and OS in the brain and immune responses, likely mediated by changes in gut flora. Therefore, this study accentuates the critical importance of accurately reporting the housing conditions of laboratory animals to refine our interpretation of experimental data and prevent inconsistencies. Detailed reporting is essential to enhance the translatability of preclinical models, which is crucial for the evaluation of AD therapeutics.

### Supplementary Information

Below is the link to the electronic supplementary material.

Supplementary file1 (DOCX 192 kb)

## Data Availability

The datasets generated and/or analyzed during the current study are available from the corresponding author upon reasonable request. Materials used in the study, and experimental protocols are also available upon request.
