# Supplementary material for: Vivaria housing conditions expose sex differences in brain oxidation, microglial activation, and immune system states in aged hAPOE4 mice
Source: Exp Brain Res. 2024 Jan 11;242(3):543–57. doi: 10.1007/s00221-023-06763-x (PMC10894770; doi:10.1007/s00221-023-06763-x)
Supplement: Supplementary file 1 — Supplementary file1 (DOCX 192 kb) [file 221_2023_6763_MOESM1_ESM.docx]

**Vivaria housing conditions expose sex differences in brain oxidation, microglial activation, and immune system states in aged hAPOE4 mice.**

Reyes-Reyes EM^1^, Brown J^1^, Trial MD^1^, Chinnasamy D^1^, Wiegand JP^1^, Bradford D^1,2^, Brinton RD^1,2^, Rodgers KE^1*^

^1^Center for Innovation in Brain Science, University of Arizona, Tucson, AZ, USA

^2^Department of Pharmacology, College of Medicine, University of Arizona, Tucson, AZ, USA

**Supporting Information**


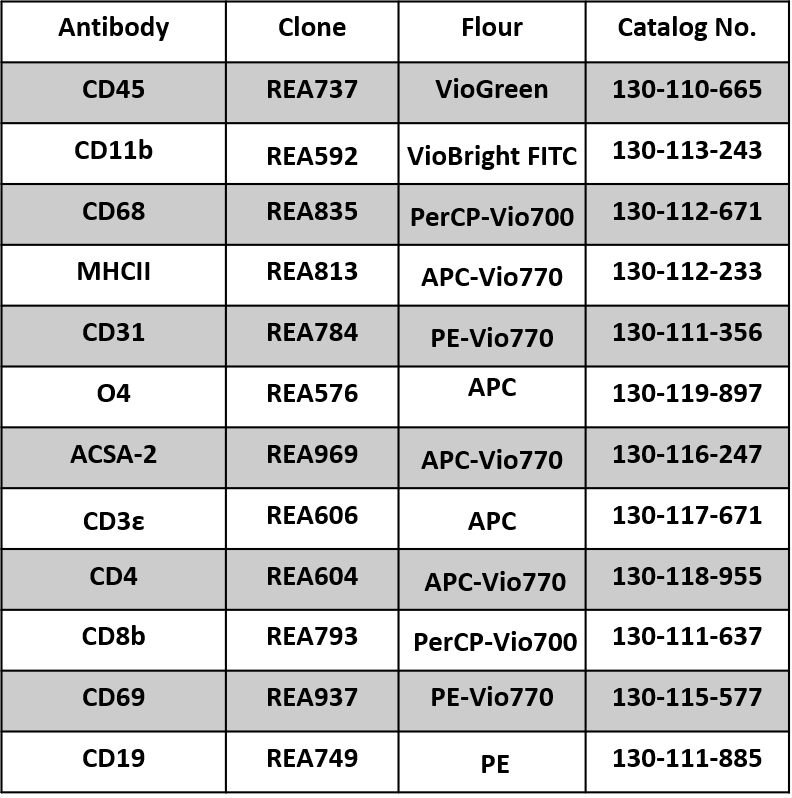


**Table 1. Antibodies**

**Table 2. Two-way ANOVA Bonferroni's multiple comparisons test**


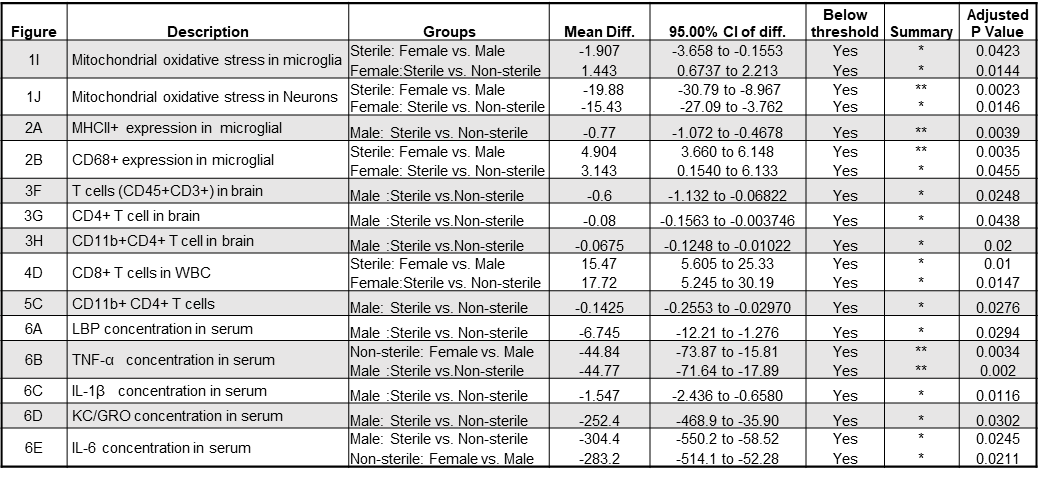


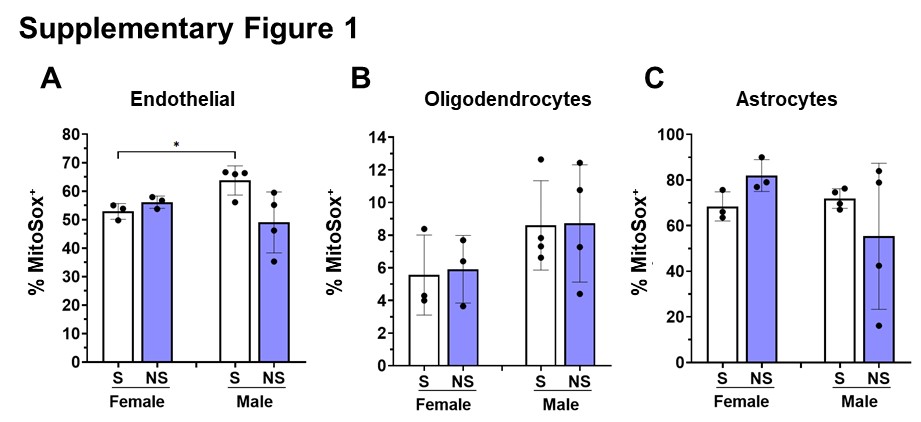


Supplementary Figure 1. Effect of housing and status on oxidant production in oligodendrocytes and astrocytes of hAPOE4 female and male. Brain cells were dissociated and s ined for type-specific civic markers and analyzed by flow cytometry. The gating strategy for the brain cell type characterization can be found in panels A-H. Oligodendrocytes and astrocytes were used for gating mitochondrial oxidative stress (MitoSox^+^). Graphs show the percentage of MitoSox^+^ endothelial cells (A), oligodendrocytes (B), and astrocytes (C). Data are represented as mean ± SD.


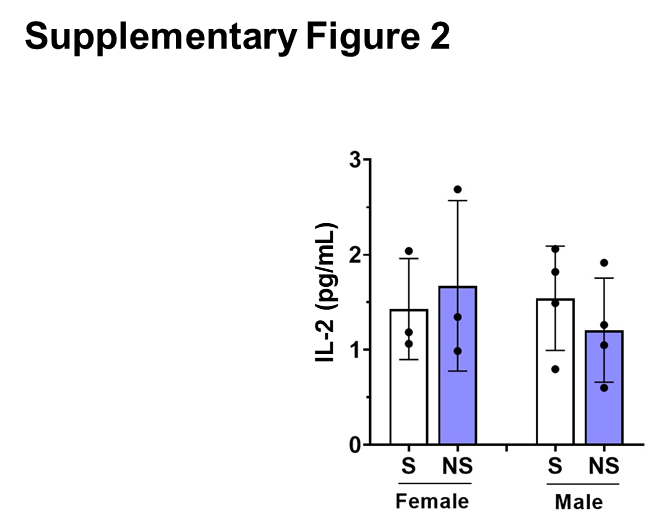


Supplementary Figure 2. The concentration of circulating IL-2 was measured from plasma collected at necropsy. Data are represented as mean ± SD.
